# Incidence and outcomes of acute kidney injury in extremely-low-birth-weight infants

**DOI:** 10.1371/journal.pone.0187764

**Published:** 2017-11-06

**Authors:** Chien-Chung Lee, Oi-Wa Chan, Mei-Yin Lai, Kai-Hsiang Hsu, Tai-Wei Wu, Wai-Ho Lim, Yu-Cheng Wang, Reyin Lien

**Affiliations:** 1 Division of Neonatology, Department of Pediatrics, Chang Gung Memorial Hospital, School of Medicine, Chang Gung University, Taoyuan, Taiwan, ROC; 2 Graduate Institute of Clinical Medical Sciences, School of Medicine, Chang Gung University, Taoyuan, Taiwan, ROC; 3 Division of Pediatric Critical Care Medicine, Department of Pediatrics, Chang Gung Memorial Hospital, School of Medicine, Chang Gung University, Taoyuan, Taiwan, ROC; 4 Department of Pediatrics, Ton-Yen General Hospital, Hsinchu, Taiwan, ROC; The University of Manchester, UNITED KINGDOM

## Abstract

**Background:**

Acute kidney injury (AKI) is a common event in the neonatal intensive care unit (NICU), especially in extremely-low-birth-weight (ELBW) infants. This cohort study investigated the incidence of and risk factors for AKI in ELBW infants and their overall survival at the postmenstrual age (PMA) of 36 weeks.

**Methods:**

All ELBW infants admitted to our NICU between January 2010 and December 2013 were enrolled. Those who died prior to 72 hours of life, had congenital renal abnormality, or had only one datum of the serum creatinine (SCr) level after the first 24 hours of life were excluded. The criteria used for the diagnosis of AKI was set according to the modified neonatal KDIGO AKI definition.

**Results:**

AKI occurred in 56% of 276 infants. Specifically, stage 1, stage 2, and stage 3 AKI occurred in 30%, 17%, and 9% of ELBW infants, respectively. High-frequency ventilation support (adjusted odds ratio [OR]: 3.4, 95% confidence interval [CI]: 1.78–6.67, p< 0.001), the presence of patent ductus arteriosus (adjusted OR: 4.3, 95% CI: 2.25–8.07, p < 0.001), lower gestational age (adjusted OR for gestational age: 0.7, 95% CI: 0.58–0.83, < 0.001), and inotropic agent use (adjusted OR: 2.6, 95% CI: 1.31–5.21, p = 0.006) were independently associated with AKI. Maternal pre-eclampsia was a protective factor (adjusted OR: 0.4, 95% CI: 0.14–0.97, p = 0.044). Infants with AKI had higher mortality before the PMA of 36 weeks with an adjusted hazard ratio (HR) of 5.34 (95% CI: 1.21–23.53, p = 0.027). Additionally, infants with stage 3 AKI had a highest HR of 10.60, 95% CI: 2.09–53.67, *p* = 0.004).

**Conclusion:**

AKI was a very common event (56%) in ELBW infants and was associated with a lower GA, high-frequency ventilation support, the presence of PDA, and inotropic agent use. AKI reduced survival of ELBW infants before the PMA of 36 weeks.

## Introduction

Acute kidney injury (AKI) is a common condition in critically ill infants in the neonatal intensive unit (NICU), particularly in preterm infants who are susceptible to hemodynamic alternations, perinatal and nosocomial infections, and multiple nephrotoxic medications during hospitalization. Preterm infants were found to have accelerated renal maturation, a higher percentage of morphologically abnormal glomeruli, and a higher glomerular volume (which is suggestive of renal hyperfiltration). These observations suggest that the kidneys of preterm infants have fewer functional nephrons, which increase their vulnerability to impaired renal function [1]. Preterm infants with AKI may have long-lasting renal damage that can lead to glomerulosclerosis and chronic kidney disease in adult life [2–5]. In addition to long-term renal morbidities, premature infants with AKI have been found to have higher mortality than those without AKI [6–9].

However, the diagnosis of AKI in extremely premature infants is very difficult, especially during the first postnatal days of life when they are the most vulnerable to develop AKI. A serum creatinine (SCr) level of more than 1.5 mg/dL or a urine volume of less than 0.5 mL/kg/h were broadly used to identify AKI in neonates before 2008 [10]. However, neonatal SCr level immediately after birth often reflects maternal levels, and the SCr level of extremely premature infants often increases and reaches a plateau in the first days of life before decreasing thereafter [11–15]. Therefore, recent studies have utilized modifications of the Acute Kidney Injury Network (AKIN) staging system [6, 7, 9, 16–19] or the risk, injury, failure, loss, and end-stage renal disease classification (RIFLE) [20–23] for diagnosing AKI in neonates. Furthermore, Kidney Disease: Improving Global Outcomes (KDIGO), a non-profit foundation, combined both RIFLE and AKIN definitions to provide a single tool for use in both research and clinical practice in 2013.[24] For the neonatal population, Jetton and Askenazi had proposed a modified KDIGO definition for neonatal AKI (Table 1) [25]. These percentage-based definitions of neonatal AKI indicate the severity of AKI and identify different outcomes.

**Table 1:** Neonatal acute kidney injury modified from the kidney disease: Improving global outcomes serum creatinine criteria.

Stage	Serum creatinine (SCr)	Urine output
0	No change in SCr or increase < 0.3 mg/dL	≥ 0.5 mL/kg/h
1	SCr increase ≥ 0.3 mg/dL within 48 h or SCr increase ≥ 1.5–1.9 baseline SCr^a^ within 7 d	< 0.5 mL/kg/h for 6–12 h
2	SCr increase ≥ 2 to 2.9 baseline SCr ^a^	< 0.5 mL/kg/h for ≥12 h
3	SCr increase ≥ 3 baseline SCr ^a^ or SCr ≥ 2.5 mg/dL or receipt of dialysis	< 0.3 mL/kg/h for ≥ 24 h or anuria for ≥ 12 h

^a^Baseline SCr was defined as the lowest previous SCr level and was examined only after 24 hours of life in our study.

The incidence of neonatal AKI, based on the definition and method of study (S1 Table), varies from 2.5% to 17.6% [26–31] in NICU patients, and could reach up to 40% [32] in very-low-birth-weight (VLBW, BW<1500 gms.) and to 60% in extremely-low-birth-weight (ELBW, BW<1000 gms.) infants [33]. Risk factors for AKI in VLBW infants include lower birth weight, lower gestational age (GA), lower Apgar scores (ASs), umbilical arterial catheterization, mechanical ventilation, inotropic support, nephrotoxic medications, and higher illness severity at admission [6, 7, 32, 34]. Although ELBW infants are most vulnerable to AKI, the incidence and impact of AKI in such infants remain unclear. In response, this study investigated the incidence of and risk factors for AKI in ELBW infants and examined their effect on overall survival at the postmenstrual age (PMA) of 36 weeks. This study confirmed that AKI is common in ELBW infants and it could affect their survival. Four risk factors and one protective factor were identified.

## Patients and methods

This retrospective cohort study was conducted in the NICU of Chang Gung Memorial Hospital between January 2010 and December 2013. The unit is a referral center in northern Taiwan with 37 level III-VI intensive care beds. We enrolled all ELBW infants admitted to the unit during this study period with the exclusion of those who did not survive until 72 hours of postnatal life, had any significant congenital abnormality of the kidney, or had only one SCr datum. The SCr levels of all infants were examined at 24 hours of age, and then weekly in our NICU. For critical infants, the SCr level was checked every 1 to 2 days if necessary. This study was approved by the Institutional Review Board of Chang Gung Memorial Hospital with an approval number: 104-3966B. The IRB waived the requirement for informed consent for this retrospective cohort study. However, the study was performed in accordance with the Declaration of Helsinki.

### AKI definition

AKI was diagnosed on the basis of changes in the SCr level according to the modified neonatal AKI KDIGO definition (Table 1). AKI was defined as an increase in the SCr level by ≥0.3 mg/dL within 48 hours or ≥1.5 times from the baseline within 7 days. Newborns are unique in that the SCr level immediately after birth often reflects maternal levels. Studies have reported that the mean SCr level in preterm infants rises during the first two days of postnatal life, reaches a plateau for a few days, and then decreases thereafter [11, 12, 14, 25, 35]. Therefore, we defined the baseline SCr level as the lowest previous SCr level after 24 hours of age. Because this is a retrospective study and the urine output is hard to trace as detailed as per kilogram per hour from medical records, we did not include urine output criteria.

### Data abstraction

Maternal demographic data were collected, including the diagnoses of diabetes, preeclampsia, placental hemorrhage, premature rupture of membranes (PROM) for more than 24 hours, and chorioamnionitis (whether suspected by obstetricians or confirmed by amniotic fluid cultures), and the use of antibiotics and antenatal steroids. Infant demographic data were also collected, including GA, birth weight, sex, delivery mode, ASs at 1 and 5 minutes, and pH value of the first arterial blood analysis; we also identified infants who were part of a multiple birth. The clinical characteristics of preterm care were documented, including the insertion of an umbilical catheter, high-frequency ventilation support, and the use of medications such as inotropic agents, gentamicin, or NSAIDs. Comorbid conditions were also reviewed, such as culture-proven sepsis (excluding coagulase-negative staphylococci) or fungemia, presence of a patent ductus arteriosus (PDA) confirmed by echocardiogram, PDA ligation, intraventricular hemorrhage (IVH), and necrotizing enterocolitis (Bell’s stage II or III). Because prolonged mechanical ventilation due to moderate or severe bronchopulmonary dysplasia (BPD) is a well-known risk of mortality [36], we chose the primary outcome as survival up to postmenstrual age (PMA) of 36 weeks. Discharge before the PMA of 36 weeks was regarded as survival. Since all ELBW infants were born at less than 32 weeks gestational age, the definition of moderate or severe BPD was oxygen dependency at 36 weeks PMA. “BPD” mentioned in this text was referred to moderate or severe BPD, and we used the composite outcome of death prior to discharge or BPD as a secondary outcome. We also selected total hospitalization days, and BPD at 36 weeks PMA as other secondary outcomes.

### Statistical analysis

The Kolmogorov–Smirnov test was used to assess the normality of the distribution of investigated parameters. All continuous variables were not distributed normally and are expressed as the median and interquartile range (IQR). Differences were then examined using the Mann–Whitney *U* test, and categorical variables were analyzed using the Pearson chi-square test or Fisher’s exact test.

To identify independent risk factors for AKI, demographic data and clinical characteristics were compared between infants with and without AKI. Variables with a *p* value of <0.05 in the univariate analysis were incorporated into a multivariate logistic regression model with backward analysis to explore the independent risk factors of AKI. The collinear variables and the variables with less than 10 patients of the smallest group were excluded from multivariate logistic regression. To Cox regression was also performed for all possible predictors of mortality. Again, variables with a *p* value of <0.05 in the univariate model were incorporated into the multivariate model with backward analysis to calculate the hazard ratio (HR) of mortality for infants with AKI. Statistical analysis was performed using SPSS Statistics, version 22.0 (Armonk, NY: IBM Corp).

## Results

### Incidence of AKI

**Fig 1:**
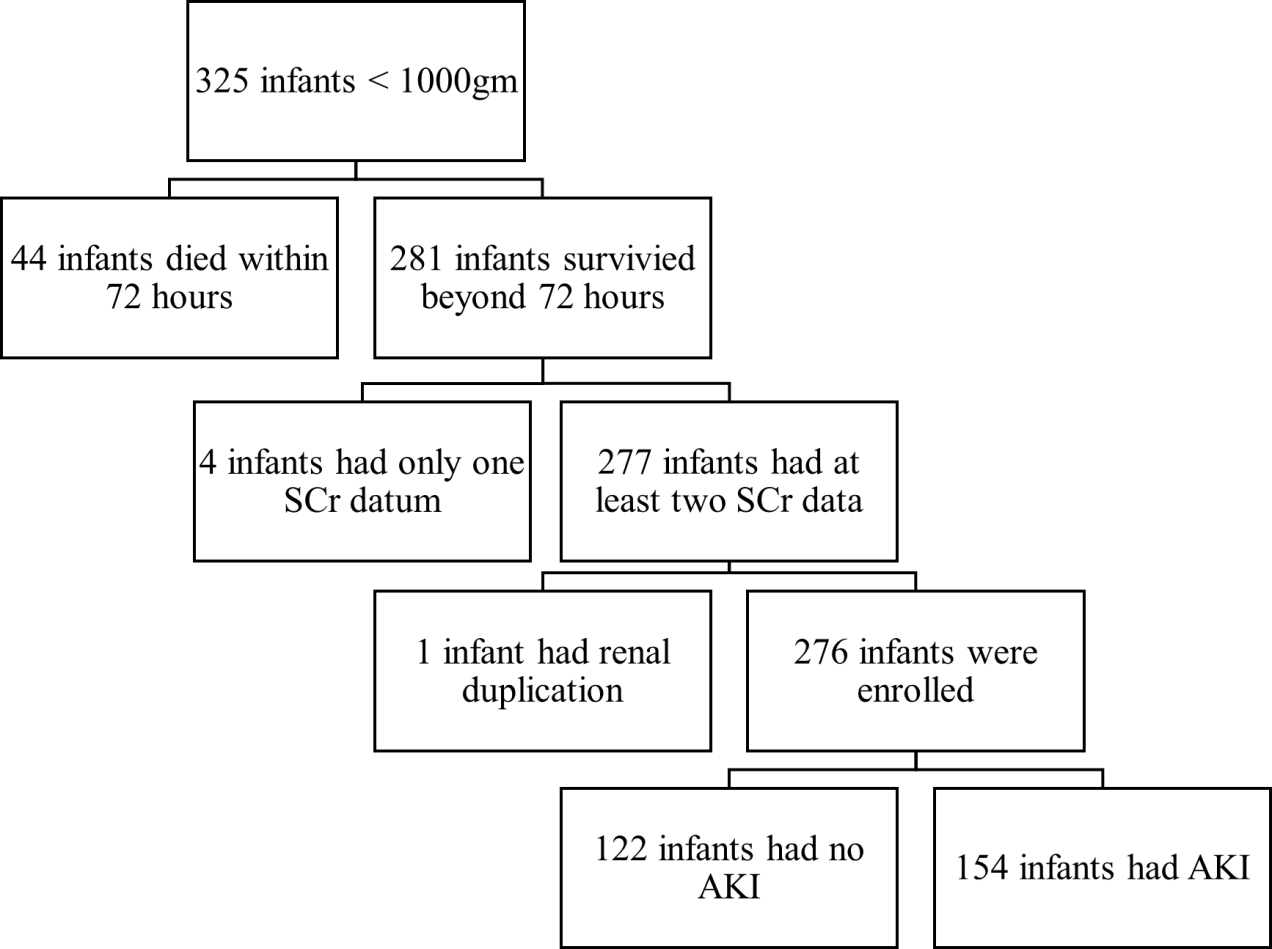
Flowchart of the selection of patients.

Of the 325 ELBW preterm infants who were admitted to our NICU, 44 were excluded due to death within the first 72 postnatal hours, 4 were excluded due to inadequate SCr data (≤1 value after 24 hours of age), and 1 was excluded due to renal duplication (Fig 1). Of the 276 enrolled infants, 154 (56%) had AKI, of which 82 (30%) had stage 1 AKI, 47 (17%) had stage 2 AKI, and 25 (9%) had stage 3 AKI. The infants with a lower GA or lower birth weight were more prone to develop AKI (Tables 2 and 3, p < 0.001). Except for two infants who developed stage 3 AKI after 1 month of age (47 days and 58 days of age, respectively), all infants developed AKI between 2 and 18 days of life (median: 4 days, IQR: 3–6 days).

**Table 2:** Incidence of AKI stratified by gestational age.

AKI	23 wks, n = 11	24–25 wks, n = 106	26–27 wks, n = 88	28–29 wks, n = 48	≥30 wks, n = 23	Total n = 276	p
No	2 (18%)	19 (18%)	48 (55%)	34 (71%)	19 (83%)	122 (44%)	<0.001
Stage 1	2 (18%)	46 (43%)	22 (25%)	8 (17%)	4 (17%)	82 (30%)	
Stage 2	4 (36%)	27 (26%)	15 (17%)	1 (2%)	0	47 (17%)	
Stage 3	3 (27%)	14 (13%)	3 (3%)	5 (10%)	0	25 (9%)	

**Table 3:** Incidence of AKI stratified by birth weight.

AKI	≤500 g, N = 9	501–750 g, N = 106	751–1000 g, N = 161	Total N = 276	p
No	1 (11%)	34 (32%)	87 (54%)	122 (44%)	<0.001
Stage 1	5 (56%)	33 (31%)	44 (27%)	82 (30%)	
Stage 2	0	26 (25%)	21 (13%)	47 (17%)	
Stage 3	3 (33%)	13 (12%)	9 (6%)	25 (9%)	

### Risk factors for AKI

The demographic data and clinical characteristics of the infants with and without AKI are listed in Table 4. The infants with AKI had a lower GA, lower birth weight, lower 1- and 5-minute Apgar scores and lower first arterial blood pH. The infants with AKI were more likely to have umbilical artery catheters, require high-frequency ventilation support, and use inotropic agents. Furthermore, neonatal AKI was associated with several maternal conditions. Infants born to mothers with diabetes, hypertension, or preeclampsia, and infants delivered by cesarean section seemed to be protected from neonatal AKI. On the other hand, maternal infection-associated variables such as chorioamnionitis and prenatal antibiotic use were risk factors to neonatal AKI. The infants with AKI were also associated with comorbidities, namely PDA and IVH. However, after controlling for confounding variables (Table 5), only four risk factors were determined to be associated with AKI, namely high-frequency ventilation support, the presence of PDA, a lower GA and, inotropic agent use while one protective factor, maternal pre-eclampsia, was found.

**Table 4:** Demographic data and clinical characteristics of infants with and without AKI.

	No AKI, n = 122	AKI, n = 154	*p* value
Infants’ demographic data			
Gestational age (weeks)*	27 (26,28)	25 (24,26)	<0.001
Birth weight*	847 (741,915)	745 (662,832)	<0.001
Male gender	56 (46%)	88 (57%)	0.063
Cesarean section	98 (80%)	104 (68%)	0.015
Multiple birth	27 (22%)	43 (28%)	0.272
Apgar score at 1 minute*	5 (4,7)	4 (3,5)	<0.001
Apgar score at 5 minutes*	8 (7,9)	7 (6,8)	<0.001
1st arterial blood gas pH*	7.33 (7.27,7.39)	7.28 (7.18,7.36)	<0.001
Clinical intervention			
Umbilical artery catheter	82 (67%)	121 (79%)	0.034
High frequency ventilator	51 (42%)	125 (81%)	<0.001
Gentamicin	88 (72%)	96 (62%)	0.087
NSAIDs	7 (6%)	15 (10%)	0.223
Inotropic agents	66 (54%)	132 (86%)	<0.001
Co-morbidity			
Culture-proven bacteremia/fungemia	24 (20%)	34 (22%)	0.626
Intraventricular hemorrhage	25 (21%)	62 (40%)	0.001
Necrotizing enterocolitis, stage II/III	10 (8%)	6 (4%)	0.129
Patent ductus arteriosus	44 (36%)	118 (77%)	<0.001
Maternal clinical exposure			
Diabetes	7 (6%)	2 (1%)	0.047
Hypertension	12 (10%)	6 (4%)	0.047
Pre-eclampsia	31 (25%)	11 (7%)	<0.001
Chorioamnionitis	10 (8%)	27 (18%)	0.024
Prenatal antibiotics	52 (43%)	85 (55%)	0.038
Prenatal steroids	83 (68%)	93 (60%)	0.190
Placental hemorrhage	10 (8%)	21 (14%)	0.155
Premature rupture of membranes	43 (35%)	51 (33%)	0.711

*presented as the median and interquartile range.

**Table 5:** Independent risk factors for AKI.

	Adjusted odds ratio	p value
High frequency ventilator	3.4 (1.78–6.67)	<0.001
Patent ductus arteriosus	4.3 (2.25–8.07)	<0.001
Gestational age	0.7 (0.58–0.83)	<0.001
Inotropic agents	2.6 (1.31–5.21)	0.006
Pre-eclampsia	0.4 (0.14–0.97)	0.044

Adjusted for gestational age, Cesarean section, Apgar scores at 1 minutes, first arterial blood pH, umbilical arterial catheter insertion, high-frequency ventilation support, inotropic agent use, intraventricular hemorrhage, patent ductus arteriosus, maternal pre-eclampsia, chorioamnionitis, and prenatal antibiotic use.

### AKI and survival at PMA of 36 weeks

**Fig 2:**
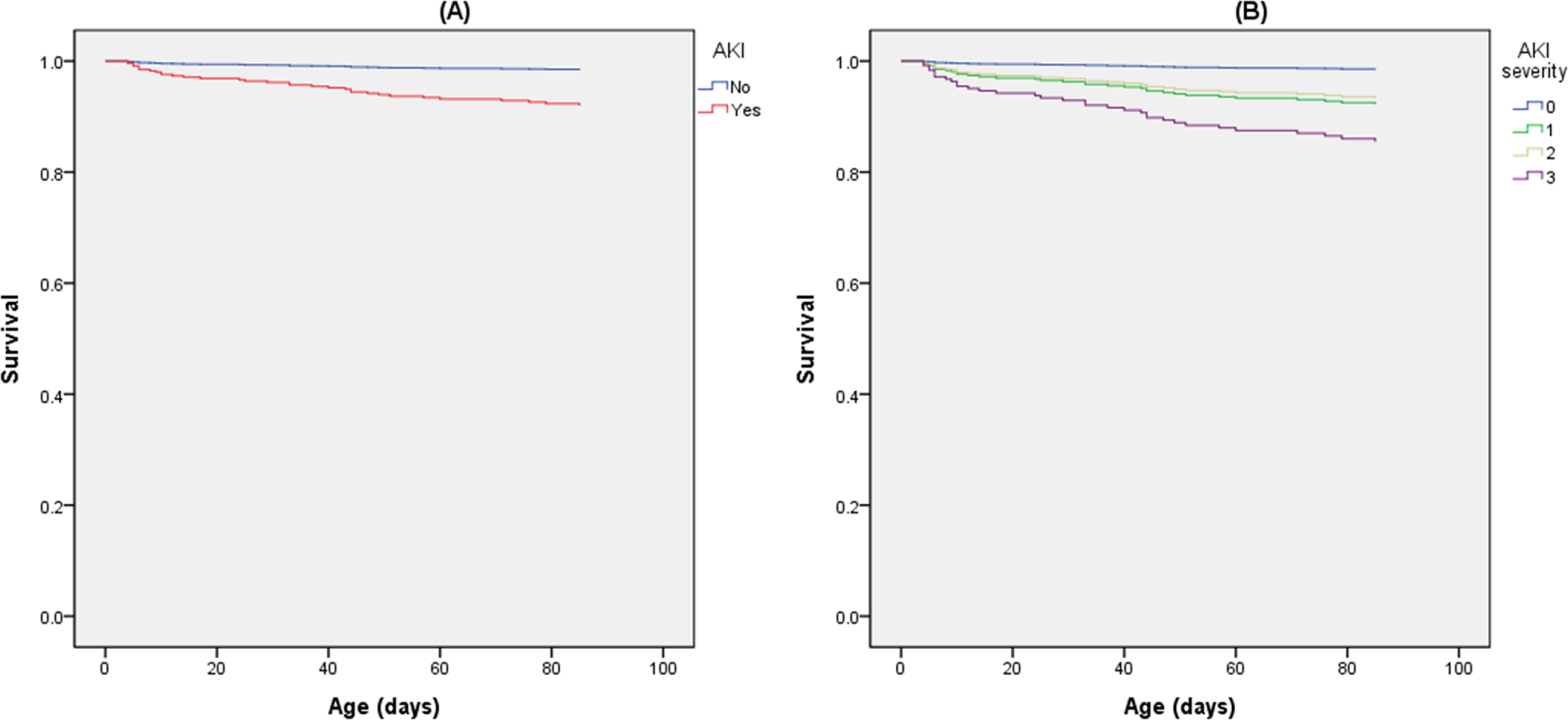
(A) Survival curve for infants with AKI. (B) Survival curve for infants with each stage of AKI, controlled for GA, Apgar scores at 1 minute, first arterial blood gas pH, IVH, high-frequency ventilation support, inotropic agent use, PDA, culture-proven bacteremia/fungemia, and PROM.

Of the 154 infants with AKI, 31 (20%) died before the PMA of 36 weeks compared with only 2 (2%) of the 122 infants without AKI (p < 0.001). All possible predictors of mortality before the PMA of 36 weeks were analyzed using a Cox proportional hazard regression univariate model (Table 6). AKI was a strong predictor of mortality before the PMA of 36 weeks with a crude HR of 13.12 (95% CI: 3.14–54.81, p < 0.001); GA and birth weight were also strong predictors of mortality (p < 0.001). Other significant predictors included the 1-minute AS, pH of the first arterial blood gas, IVH, high-frequency ventilation support, inotropic agent use, PDA, culture-proven bacteremia and/or fungemia, and PROM. After controlling for these significant variables, AKI was still a significant predictor of mortality (HR: 5.34, 95% CI: 1.21–23.53, *p* = 0.027; Table 6 and Fig 2). In particular, stage 3 AKI significantly contributed to increased mortality (adjusted HR: 10.60, 95% CI: 2.09–53.67, *p* = 0.004). Among the 243 infants who survived up to the PMA of 36 weeks, those with AKI had a longer hospitalization duration (median: 112days, IQR: 99–144 days] vs. median: 95days, IQR: 76–125 days; *p* = 0.004) and a higher rate of BPD (76% vs. 46%, *p* < 0.001). Moreover, after controlling for GA and birth weight in logistic regression, the infants with AKI still had a higher risk of BPD (adjusted OR: 2.25, 95% CI: 1.21–4.21, *p* = 0.011). In addition, infants with AKI had higher risk to develop BPD or death prior to discharge (81% vs. 48%, *p* < 0.001) with the adjusted OR of 2.74 (95% CI: 1.48–5.09, *p* = 0.001).

**Table 6:** Predictors of mortality before the PMA of 36 weeks.

	Crude hazard ratio (95% CI)*	*p* value
Univariate model		
Perinatal factors		
Gestational age (weeks)	0.60 (0.47–0.78)	<0.001
Birth weight (100g)	0.53 (0.41–0.70)	<0.001
Male gender	0.79 (0.40–1.58)	0.510
Multiple birth	0.96 (0.44–2.14)	0.927
Apgar score at 1 minute	0.80 (0.67–0.94)	0.009
Apgar score at 5 minutes	0.86 (0.73–1.01)	0.059
1^st^ arterial blood gas pH	0.10 (0.02–0.61)	0.013
Postnatal factors		
Intraventricular hemorrhage	2.12 (1.07–4.19)	0.032
Necrotizing enterocolitis, stage II/III	1.02 (0.25–4.28)	0.974
High frequency ventilator support	19.23 (2.63–140.75)	0.004
Inotropic agent use	6.44 (1.54–26.92)	0.011
Patent ductus arteriosus	2.23 (1.01–4.95)	0.048
Culture-proved bacteremia/fungemia	3.22 (1.62–6.38)	0.001
Any acute kidney injury	13.12 (3.14–54.81)	<0.001
Acute kidney injury stage 1	11.76 (2.69–51.45)	0.001
Acute kidney injury stage 2	10.81 (2.29–50.89)	0.003
Acute kidney injury stage 3	23.00 (4.88–108.33)	<0.001
Maternal factors		
Maternal diabetes	0.05 (0.00–173.29)	0.466
Maternal hypertension	1.45 (0.44–4.75)	0.540
Pre-eclampsia	0.17 (0.02–1.21)	0.076
Chorioamnionitis	0.62 (0.19–2.02)	0.424
Placental hemorrhage	0.80 (0.25–2.63)	0.717
Premature rupture of membrane	0.41 (0.17–0.99)	0.048
Multivariate model^#^		
Any acute kidney injury	5.34 (1.21–23.53)	0.027
Acute kidney injury stage 1	5.48 (1.22–24.69)	0.027
Acute kidney injury stage 2	4.68 (0.95–23.03)	0.058
Acute kidney injury stage 3	10.60 (2.09–53.67)	0.004

*Cox proportional hazard ratios for GA per week increase, Birth weight per 100 g increase, Apgar scores and first arterial blood gas pH per point increase, and categorical variables compared with any AKI, stage 1 AKI, stage 2 AKI, stage 3 AKI, and absence of AKI.

^#^Control of GA, Apgar scores at 1 minute, first arterial blood gas pH, IVH, high-frequency ventilation support, inotropic agent use, PDA, culture-proven bacteremia/fungemia, and PROM.

## Discussion

ELBW infants are very vulnerable to AKI, but there are little studies focused on AKI in this group. We are interested in exploring the incidence of AKI in ELBW infants and its impact on their overall survival at the PMA of 36 weeks. In this study, we found that AKI incidence could reach 56% in ELBW infants, and these infants with AKI have higher mortality rate before 36 weeks PMA with an adjusted HR of 5.34. The risk factors of ELBW infants to develop AKI included a lower GA, high-frequency ventilation support, presence of PDA, and inotropic agent use. Maternal pre-eclampsia was a protective factor.

The reported incidence of AKI in NICUs ranges widely depending on the patient sample and AKI definition used (S1 Table). The incidence of AKI in VLBW infants has been estimated to be 18%–40% [7, 32, 34]. Viswanathan et al. [37] reported that the incidence of AKI in ELBW infants was 12.5% (59/472) by using the traditional AKI definition (oliguria of <1 mL/kg/h after 24 hours of life or a SCr level of >1.5 mg/dL after 72 hours of life). In a prospective, landmark AKI study conducted by Koralkar et al. [7] in 2011 using the modified neonatal KDIGO definition, the incidence of AKI was 18% (41 of 229) in VLBW infants and 29% (37 of 126) in ELBW infants. Stojanović et al. [9] conducted a retrospective study using the same AKI definition and reported that the incidence of AKI was 26% (39 of 150) in all preterm infants, 39% (37 of 96) in VLBW infants, and 48% (24 of 50) in ELBW infants. However, Chowdhary et al. compared the incidence of AKI in ELBW infants by different definitions, and found that AKI incidence could be as high as 56%, 59% and 60% according to pRIFLE, AKIN and KDIGO definitions, respectively[33]. In our study using KDIGO definition, the incidence was similar to Chowdhary’s report. Incidence of AKI in these 276 ELBW infants who survived beyond 72 hours of age was 56%; specifically, stage 1, 2, and 3 AKI occurred in 30%, 17%, and 9% of ELBW infants, respectively. Our study showed that AKI in most preterm infants occurred within 1 week of life (median: 4 days, IQR: 3–6 days) as reported by previous studies [7, 9, 32].

The risk factors for AKI slightly differed between preterm and term infants. Birth asphyxia and sepsis are well-known risk factors for AKI in near-term and term infants [26–30, 38–40], whereas other risk factors, such as lower ASs, ventilation support, PDA, inotropic agent use, ibuprofen use, IVH, and umbilical arterial catheter insertion, were more common in preterm infants [6, 7, 9, 32, 41, 42]. These findings indicate that the etiologies of AKI in preterm infants are quite different from those in term infants. In our study, high-frequency ventilation support, the presence of PDA, a lower GA, and inotropic agent use were independently associated with AKI in ELBW infants. Preterm infants more easily develop AKI than term infants [7, 9, 15, 27, 30, 32]. The higher incidence of AKI in infants with a lower GA might be attributable to greater chances of exposure to other AKI risk factors. However, according to our multivariate analysis model, in which all possible risk variables were included, a lower GA was still an independent risk factor for AKI (adjusted OR for gestational age: 0.7, 95% CI: 0.60–0.92; p = 0.007). Although our study showed high-frequency ventilation support, the presence of PDA, and inotropic agent use prior to AKI were also associated with AKI as reported by previous studies, their causality should be carefully explained. Since SCr is a biomarker of kidney function and may rise late after kidney damage. In addition, severe respiratory distress requiring high-frequency ventilation support, severe hypotension requiring inotropic agents, and PDA commonly occur in the first week of life as AKI events. Therefore, kidney damage might both be a promoter and an effector of these comorbidities. Notably infant born to pre-eclampsia mother were less likely to have AKI (adjust OR: 0.4, 95% CI: 0.14–0.97, p = 0.044). The protective effect of maternal pre-eclampsia on neonatal AKI was discussed in the recent reports by Askenazi et al. [7, 43, 44]. The authors speculated that such protective effect could have arisen from prenatal ischemic pre-conditioning of the kidney, or medications and/or other interventions received for the management of maternal pre-eclampsia. Early delivery due to conditions other than pre-eclampsia, specifically fetal distress vs. pre-eclampsia, could have been another plausible explanation for this protective effect. Further well-designed, case–control studies with a unanimous AKI definition involving both functional and injury biomarkers should be conducted to clarify the causality between AKI and these associated factors.

Several studies have reported a higher mortality rate in neonates with AKI. In a matched case–control study of 195 VLBW infants conducted in 2009, Askenazi et al. [6] demonstrated that an increase of 1 mg/dL in the SCr level would increase mortality by 3.5-fold (95% CI: 1.23–9.61). In 2011, Koralkar et al. [7] reported that the adjusted HR of mortality in VLBW infants with AKI was 2.46 (95% CI: 0.95–6.04). In 2014, Carmody et al. [32] reported that the adjusted HR of mortality in VLBW infants with AKI was 4.0 (95% CI: 1.4–11.5). In 2014, Stojanović et al. [9] reported that the adjusted HR of mortality in VLBW infants with AKI was 2.22 (95% CI: 1.27–3.86). Moreover, Bruel et al. [8] conducted a study that included 1461 preterm infants with a GA of <33 weeks in 2013, and found that infants with an SCr level >1.6 mg/dL at a GA of 24–27 weeks, >1.1 mg/dL at a GA of 28–29 weeks, and >1 mg/dL at a GA of 30–32 weeks had an increased mortality rate and poor neurodevelopmental outcomes at the age of 2 years. In our cohort study, AKI reduced overall survival at a PMA of 36 weeks in ELBW infants, with an adjusted HR 5.34 (95% CI: 1.21–23.53, *p* = 0.027); notably, the adjusted HR was 10.60 (95% CI: 2.09–53.67, *p* = 0.004) in the ELBW infants with stage 3 AKI.

In our study, ELBW infants with AKI had a higher risk of BPD (adjusted OR: 2.25, 95% CI: 1.21–4.21, *p* = 0.011) and a higher mortality rate. Because most AKI events occur in the early life of ELBW infants, the mechanisms of AKI causing in-hospital morbidity and mortality later on are not well clarified. In 2015, Askenazi et al. also reported the association between AKI and BPD/mortality in a prospective study involving 122 preterm infants (BW ≤1200 gms. and/or GA <31 weeks) [44]. The authors’ suggested that kidney plays a critical role in maintaining systemic cytokines, and acute kidney injury would alter the systemic inflammation and lung physiology, leading to higher risks of BPD and mortality. The view of crosstalk between kidney and lung via cytokines and inflammation was supported by several animal studies [45–47]. AKI during hospitalization is associated with high in-hospital and long-term mortality in children and adults [48–51]. Recent studies have also shown that AKI causes not only short-term, immediately recognizable consequences (e.g., electrolyte/acid base abnormalities and fluid overload), but also long-term consequences (e.g., impaired innate immunity and chronic kidney disease) [52]. Therefore, serial follow up these preterm infants to prevent long-term complications is necessary.

There were several limitations to this study. First, this was a retrospective single-center cohort study, and the most critical infants (non-survival beyond 72 hours of age) were excluded. The incidence of AKI in ELBW infants might be underestimated. Second, the severity of AKI might be underestimated because serum creatinine level was not checked daily and the peak level might be missed. The reason for not checking SCr daily was the concern of the very small blood volume in ELBW infants. Third, there were only 2 patients died before PMA of 36 weeks in non-AKI group comparing to 31 patients who died in AKI group. However, infants with AKI was still at risk of developing BPD and /or death before discharge in this study (secondary outcomes).

In conclusion, the results of this study demonstrate that AKI is very common in ELBW infants and can reduce their overall survival at the PMA of 36 weeks (adjusted HR: 4.92). AKI is independently associated with a lower GA, high-frequency ventilation support, the presence of PDA, and inotropic agent use. To appropriately manage AKI and improve its outcomes, additional studies are required to develop reliable biomarkers for its early diagnosis and preventive strategies for AKI in ELBW infants.

## Supporting information

S1 Table Current studies of AKI in neonates. (DOCX)
