# Supplementary material for: Incidence and outcomes of acute kidney injury in extremely-low-birth-weight infants
Source: PLoS One. 2017 Nov 6;12(11):e0187764. doi: 10.1371/journal.pone.0187764 (PMC5673227; doi:10.1371/journal.pone.0187764)
Supplement: S1 Table — (DOCX) [file pone.0187764.s001.docx]

**S1 table. Current studies of AKI in neonates**

| Study | Population | AKI definition | Incidence | AKI mortality |
| --- | --- | --- | --- | --- |
| Studies adopting old definitions | | | | |
| Agras et al  2004 | NICU neonates  N=1311 | SCr >1.5 mg/dl | 45/1311 (3.4%) | 11/45 (24%) |
| Lunn et al  2006 | NICU neonates  N=457 | SCr > 100 μmol/L  at 48 h of age | 41/457 (8.8%) | 10/41 (24%) |
| Mortazavi et al 2009 | NICU neonates  N=6042 | SCr > 1.5 mg/dl or BUN > 20mg/dL | 151/6042 (2.5%) | 31/151 (20.5%) |
| Vachvanich-sanong  et al 2012 | NICU neonates  N=139 AKI | SCr ≥ 2 mg/dl  or SCr ≥ 2 times trough level | 1984–1995: 0.9%  1996–2001: 4.5%  2002–2007: 6.3% | 54/139 (39%) |
| Bolat et al  2013 | NICU neonates  N=1992 | SCr after 48 h of age  SCr > 1.5 mg/dL  or increase 0.3 mg/dL per day | 168/1992 (8.4%) | 40/168 (23.8%) |
| Viswanathan et al 2012 | ELBW infants  N=472  Case - control  N=46 - 46 | SCr > 1.5 mg/dL after 72 h of age  or oliguria of < 1 ml/kg/h after 24 h of age | 59/472 (12.5%) | 33/46 (70%) |
| Studies adopting new percentage-based definitions (AKIN or pRIFLE or KDGIO) | | | | |
| Askenazi et al 2009 | VLBW infants  N=195 | SCr since birth,  Neonatal AKIN | Matched case-control | 1 mg/dl rise in SCr →  3.5-fold death (adjusted OR 3.44, 95% CI 1.23 – 9.61) |
| Koralkar et al 2011 | VLBW infants  N=229 | SCr since birth  Neonatal AKIN | 41/229 (18%)  Stage 1: 4%  Stage 2: 4%  Stage 3: 9% | 17/41 (42 %)  Adjust HR 2.46 (95% CI, 0.95–6.04) |
| Bezerra et. al. 2013 | NICU neonates  N=312 | SCr after 48 h of age  pRIFLE | 54/312 (17.3%) | Reduced UO (< 1.5ml/kg/)  Adjust OR 3.8 (95% CI, 1.3–10.6) |
| Rhone et al  2014 | VLBW infants  N=107 | SCr since birth  Neonatal KDIGO  (exclude death case) | 28/107 (26.2%)  Stage 1: 20%  Stage 2: 6%  Stage 3: 1% | No data |
| Carmody et al 2014 | VLBW infants  N=455 | SCr since birth  Neonatal KDIGO | 181/455 (39.8%)  Stage 1: 26%  Stage 2: 11%  Stage 3: 3% | 35/181 (19%)  Adjust OR 4.0 (95% CI, 1.4–11.5) |
| Stojanović et al 2014 | Preterm  N=150 | SCr after 48 h of age  Neonatal AKIN | 39/150 (26%)  Stage 1: 13%  Stage 2: 4%  Stage 3: 9% | 27/39 (69%)  Adjust HR 2.215 (95% CI, 1.27-3.86) |
| Daga et al  2017 | VLBW infants  N=115 | SCr after 48 h of age  AKIN and pRIFLE | AKIN: 20.1%  pRIFLE: 22.6% | No data |
| Chowdhary et al.  2017 | ELBW infants  N=483 | SCr after 72 h of age  AKIN, pRIFLE and neonatal KDIGO | pRIFLE: 56%  AKIN: 59%  KDIGO: 60% | pRIFLE: 66/268 (25%)  AKIN: 68/285 (24%)  KDIGO: 68/291 (23%) |
| Our study 2015 | ELBW infants  N=276 | SCr after 24 h of age  Neonatal KDIGO | 154/276 (56%)  Stage 1: 30%  Stage 2: 17%  Stage 3: 9% | 31/154 (20%)  Adjust HR 4.92 (95% CI, 1.04 - 23.38) |
